# Supplementary figures and images for: Mapping the architecture of the initiating phosphoglycosyl transferase from S. enterica O-antigen biosynthesis in a liponanoparticle
Source: eLife. 2024 Feb 15;12:RP91125. doi: 10.7554/eLife.91125 (PMC10942596; doi:10.7554/eLife.91125)

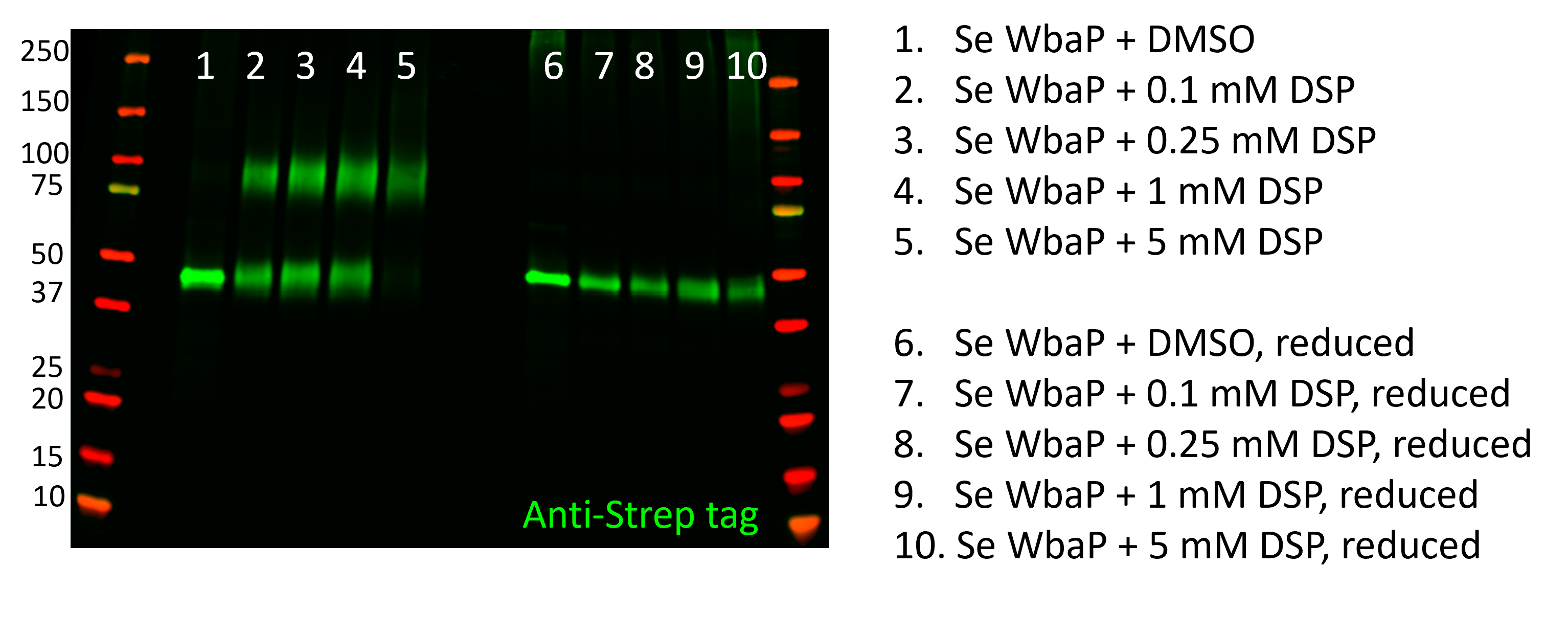

Supplement: Figure 3—source data 1. [file elife-91125-fig3-data1.zip › figure_3D_labeled_uncropped.png]

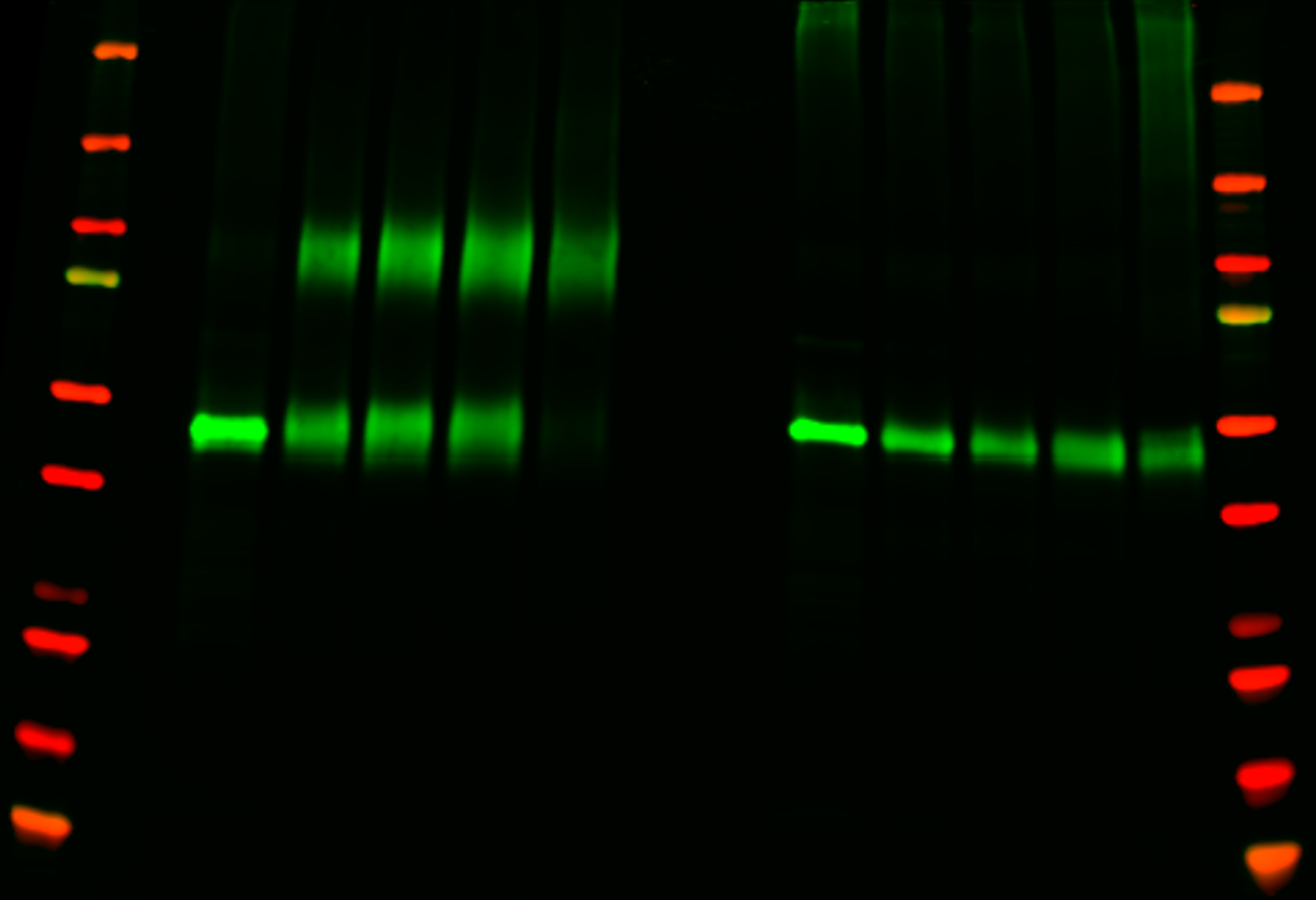

Supplement: Figure 3—source data 1. [file elife-91125-fig3-data1.zip › Figure_3D_unlabeled.tif]

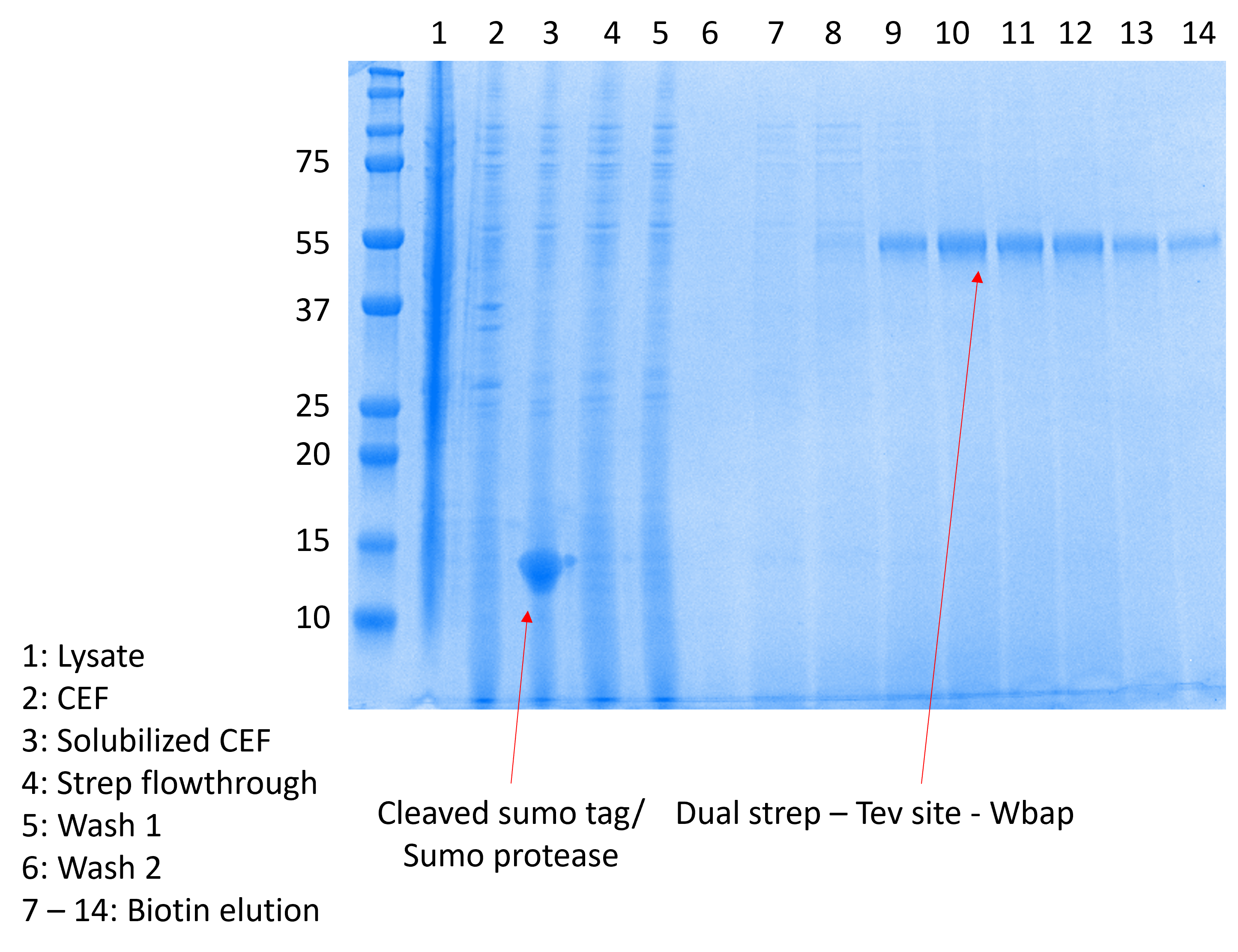

Supplement: Figure 3—figure supplement 1—source data 1. [file elife-91125-fig3-figsupp1-data1.zip › figure_3-figure_supplement_1A_labeled.png]

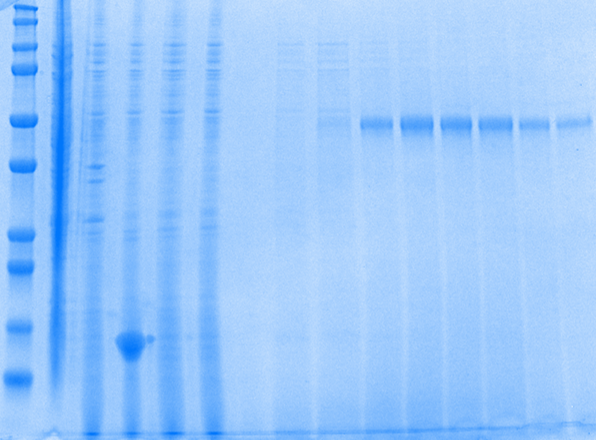

Supplement: Figure 3—figure supplement 1—source data 1. [file elife-91125-fig3-figsupp1-data1.zip › figure_3-figure_supplement_1A_unlabeled.png]

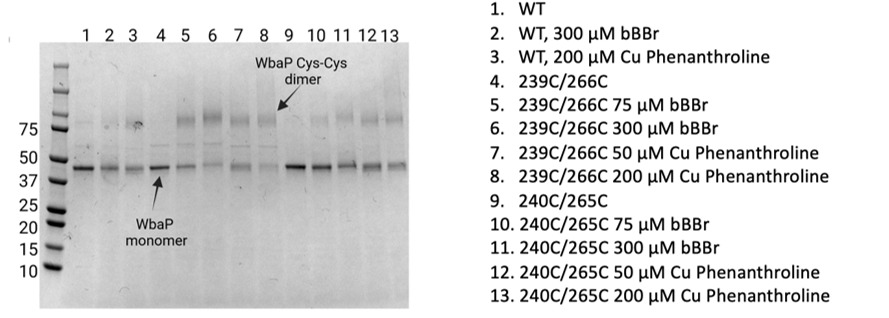

Supplement: Figure 5—source data 1. [file elife-91125-fig5-data1.zip › Figure_5D_uncropped.png]

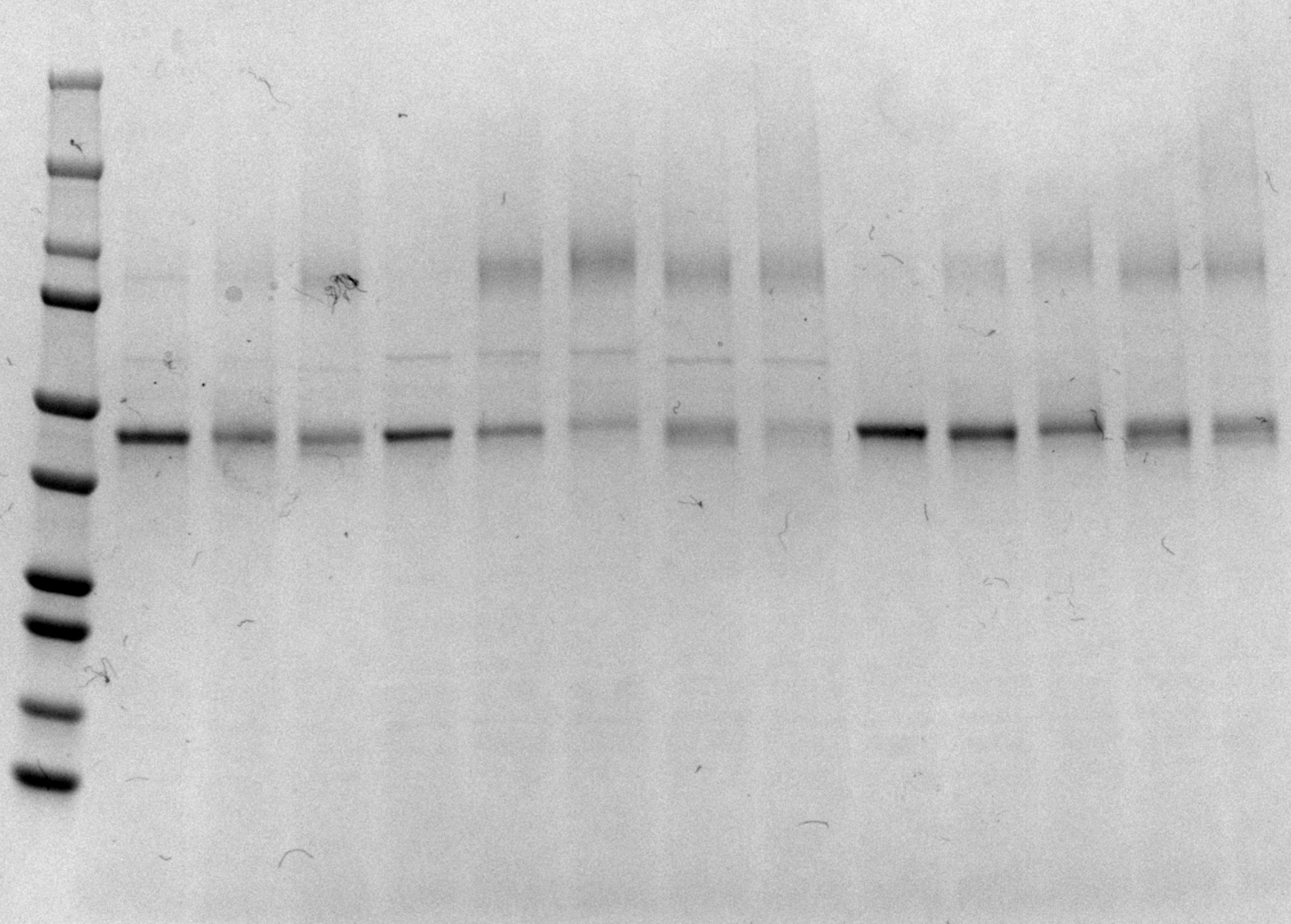

Supplement: Figure 5—source data 1. [file elife-91125-fig5-data1.zip › Figure_5D_unlabeled.tif]

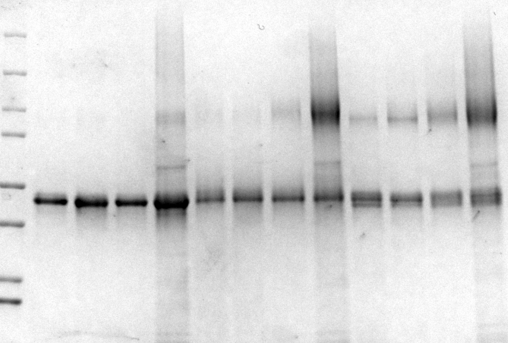

Supplement: Figure 5—source data 2. [file elife-91125-fig5-data2.zip › Fig_5E_unlabled.tif]

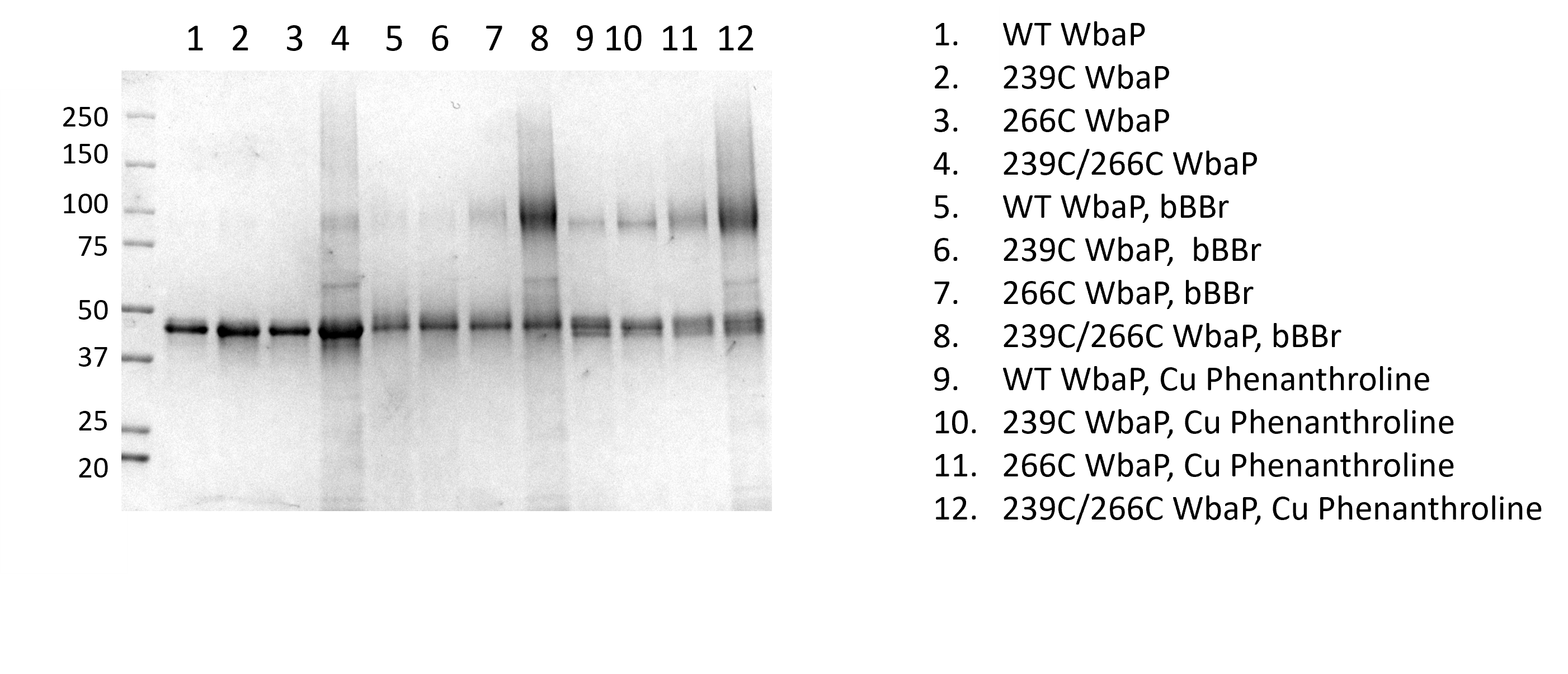

Supplement: Figure 5—source data 2. [file elife-91125-fig5-data2.zip › figure_5E_uncropped.png]

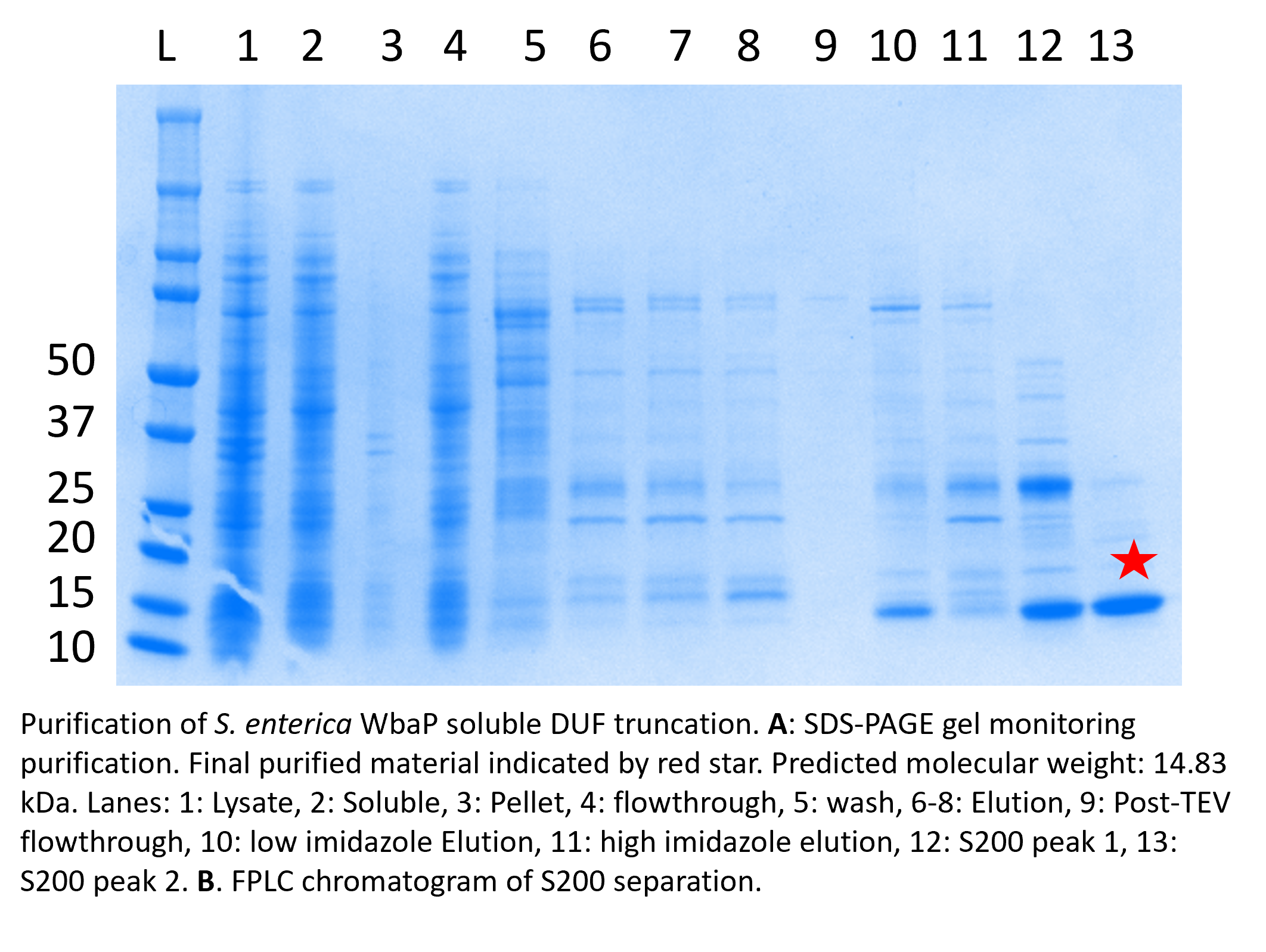

Supplement: Figure 9—figure supplement 1—source data 1. [file elife-91125-fig9-figsupp1-data1.zip › figure_9-figure_supplement_1__labeled.png]

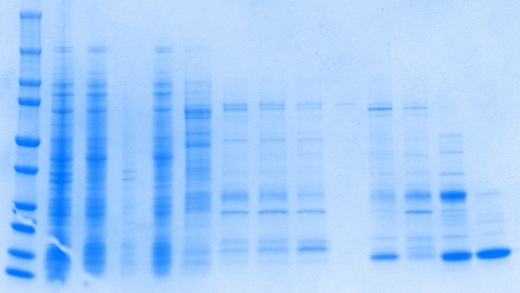

Supplement: Figure 9—figure supplement 1—source data 1. [file elife-91125-fig9-figsupp1-data1.zip › figure_9-figure_supplement_1_unlabeled.png]
