# Supplementary material for: Mapping the architecture of the initiating phosphoglycosyl transferase from S. enterica O-antigen biosynthesis in a liponanoparticle
Source: eLife. 2024 Feb 15;12:RP91125. doi: 10.7554/eLife.91125 (PMC10942596; doi:10.7554/eLife.91125)
Supplement: Supplementary file 1. — (A) Screening chemical crosslinkers for the detection of S. enterica WbaP oligomers. S. enterica WbaP in styrene-maleic acid liponanoparticle (SMALP) was reacted with a panel of lysine-reactive crosslinkers with variable chemistries, solubilities, and lengths. Samples were analyzed by Western blot to detect the presence of crosslinked oligomers. Crosslinking efficiency was annotated as high (>30% total protein crosslinked to dimer), moderate (<30% total protein crosslinked to dimer), or none (no protein crosslinked to dimer). (B). Cryo-EM data collection, refinement, and validation statistics. (C) NanoDSF nucleotide ligand screen for soluble WbaP DUF truncation. [file elife-91125-supp1.docx]

**Supporting Information**

**Mapping the architecture of the initiating phosphoglycosyl transferase from *S. enterica* O-antigen biosynthesis in a liponanoparticle.**

Greg J. Dodge,^a^ Alyssa J. Anderson,^a^ Yi He^b^, Weijing Liu^b^, Rosa Viner^b^, Barbara Imperiali^a,^*

^a^Department of Biology and Department of Chemistry, Massachusetts Institute of Technology, Cambridge, MA 02139, USA.

^b^Thermo Fisher Scientific, San Jose CA 95134, USA.

*Corresponding author: [imper@mit.edu](mailto:imper@mit.edu)

**ORCID IDs:**

BI: 0000-0002-5749-7869

RV: 0000-0003-0550-5545

WJ: 0000-0003-2322-0500

YH: 0000-0002-5565-5579

AJA: 0000-0003-4871-4283

GJD: 0000-0002-6555-8350

Supplementary file 1A

| **Crosslinker** | **Chemistry** | **Membrane permeable** | **Water soluble** | **Spacer Length (Å)** | ***Se* WbaP crosslinking efficiency** |
| --- | --- | --- | --- | --- | --- |
| **DMA** | **imidoester** | **yes** | **yes** | **8.6** | **none** |
| **DMS** | **imidoester** | **yes** | **yes** | **11** | **none** |
| **BSOCOES** | **NHS ester** | **yes** | **no** | **13** | **moderate** |
| **DSP** | **NHS ester** | **yes** | **no** | **12** | **high** |
| **DTSSP** | **NHS ester** | **no** | **yes** | **12** | **high** |

Supplementary file 1B

|  | *S. enterica* WbaP  in SMALP #1  (EMD-41042)  (PDB 8T53) |
| --- | --- |
| **Data collection and processing** |  |
| Microscope / Detector | Krios G3i, K3 |
| Magnification | 105,000X |
| Voltage (kV) | 300 |
| Electron exposure (e–/Å^2^) | 50.13 |
| Defocus range (μm) | 0.75 - 2.5 |
| Pixel size (Å) | 0.87 |
| Total movies (no.) | 4,841 |
| Symmetry imposed | C2 |
| Initial particle images (no.) | 1,266,538 |
| Final particle images (no.) | 196,663 |
| Map resolution (Å)  FSC threshold | 4.28  0.143 |
| Map resolution range (Å) |  |
|  |  |
| **Refinement** |  |
| Initial model used | *Se* WbaP AlphaFold dimer model |
| Model composition  Non-hydrogen atoms  Protein residues  Ligands | 6660  796  0 |
| *B* factors (Å^2^)  Protein (min/max/mean)  Ligand | 65.79/728.40/310.78  n/a |
| R.m.s. deviations  Bond lengths (Å)  Bond angles (°) | 0.004  0.944 |
| Validation  MolProbity score  Clashscore  Poor rotamers (%) | 1.59  11.81  0 |
| Ramachandran plot  Favored (%)  Allowed (%)  Disallowed (%) | 98.19  1.81  0 |
|  |  |
| **Model vs Data** |  |
| CC (mask) | 0.45 |
| CC (box) | 0.45 |
| CC (peaks) | 0.23 |
| CC (volume) | 0.45 |

Supplementary file 1C

| **Sample** | **Ligand** | **Ligand Concentration** | **TM °C** | **ΔTM** |
| --- | --- | --- | --- | --- |
| Blank | None | N/A | 48.35 ± 21 |  |
| AcCoA | acetyl coenzyme A | 200 μM | 47.95 ± 0.17 | -0.4 |
| AMP | adenosine 5'-monophosphate | 200 μM | 48.10 ± 0.07 | -0.25 |
| ATP | adenosine 5'-triphosphate | 200 μM | 47.98 ± 0.08 | -0.37 |
| CMP | cytidine 5'-monophosphate | 200 μM | 48.11 ± 0.30 | -0.24 |
| CoA | coenzyme A | 200 μM | 48.02 ± 0.05 | -0.33 |
| CTP | cytidine 5'-triphosphate | 200 μM | 48.14 ± 0.35 | -0.21 |
| dTTP | 2'-deoxythymidine 5'-triphosphate | 200 μM | 48.86 ± 0.21 | 0.51 |
| FAD | flavin adenine dinucleotide | 200 μM | 49.06 ± 0.18 | 0.71 |
| FMN | flavin mononucleotide | 200 μM | 48.35 ± 0.09 | 0 |
| GDP | guanosine 5'-diphosphate | 200 μM | 48.19 ± 0.04 | -0.16 |
| GTP | guanosine 5'-triphosphate | 200 μM | 48.00 ± 0.28 | -0.35 |
| NAD | nicotinamide adenine dinucleotide | 200 μM | 48.04 ± 0.01 | -0.31 |
| NADH | nicotinamide adenine dinucleotide | 200 μM | 48.54 ± 0.09 | 0.19 |
| NADP | nicotinamide adenine dinucleotide phosphate | 200 μM | 48.11 ± 0.04 | -0.24 |
| NADPH | nicotinamide adenine dinucleotide phosphate | 200 μM | 48.05 ± 0.26 | -0.3 |
| TMP | thymidine 5'-monophosphate | 200 μM | 48.40 ± 0.07 | 0.05 |
| UDP | uridine 5-diphosphate | 200 μM | 49.6 ± 0.11 | **1.25** |
| UDP-Gal | uridine 5′-(α-D-galactopyranosyl dihydrogen diphosphate) | 200 μM | 44.47 ± 0.12 | **-3.88** |
| UMP | uridine 5'-monophosphate | 200 μM | 48.44 ± 0.27 | 0.09 |
| UTP | uridine 5'-triphosphate | 200 μM | 48.41 ± 0.07 | 0.06 |
